# Prostate cancer health and cultural beliefs of black men: The Florida Prostate Cancer Disparity Project

**DOI:** 10.1186/1750-9378-6-S2-S10

**Published:** 2011-09-23

**Authors:** Folakemi T Odedina, Getachew Dagne, Shannon Pressey, Oladapo Odedina, Frank Emanuel, John Scrivens, R Renee Reams, Angela Adams, Margareth LaRose-Pierre

**Affiliations:** 1 University of Florida, College of Pharmacy, Gainesville Florida, USA; 2 University of South Florida, College of Public Health, Tampa Florida, USA; 3 Florida Black Living Navigator, Tampa Florida, USA; 4 Florida A&M University, College of Pharmacy & Pharmaceutical Sciences, Tallahassee Florida, USA; 5 Central Florida Pharmacy Council, Orlando Florida, USA

## Abstract

**Background:**

Since behavioral factors are significant determinants of population health, addressing prostate cancer (CaP)-related health beliefs and cultural beliefs are key weapons to fight this deadly disease. This study investigated the health beliefs and cultural beliefs of black men relative to CaP, and the key socio-demographic correlates of these beliefs.

**Methods:**

The study design was a cross-sectional survey of 2,864 Florida black men, age 40 to 70, on their perceived susceptibility, perceived severity, attitude, outcomes beliefs, perceived behavioral control, CaP fatalism, religiosity, temporal orientation, and acculturation relative to CaP screening and prevention.

**Results:**

The men reported favorable attitude and positive outcome beliefs, but moderate perceived behavioral control, CaP susceptibility and CaP severity. They also had low level of acculturation, did not hold fatalistic beliefs about CaP, had high religious coping skills and had high future time perspective. Several demographic variables were found to be associated with health beliefs and cultural beliefs.

**Discussion:**

Our study provides rich data with regard to the health and cultural beliefs that might serve to inform the development of CaP control initiative for US-born and foreign-born black men.

## Background

Although prostate cancer (CaP) morbidity and mortality rates continue to drop among black men in the United States (US), [1,2] the decline lags behind that of white men. Black men continue to be disproportionately affected by CaP and have the highest morbidity and mortality rates for CaP. According to the American Cancer Society, [1] blacks have 1 in 5 lifetime probability of developing invasive CaP compared to 1 in 7 for whites. The lifetime probability of dying from invasive CaP is 1 in 23 for black men and 1 in 38 for white men. Black men also experience disparities relative to CaP survival with an overall 5-year survival rate of 95% for black men and 100% for white men. The Black racial/ethnic group is the only group that has not met the Healthy People 2010 goal of reducing CaP mortality rate to 28.8/100,000 by 2010. The average annual CaP death rate for black men between 2002 and 2006 was 56.3 and 23.6 for white men. [1,2] This is a difference of 32.7, with black men having CaP mortality rate 2.4 times higher than that of white men.^1,2^ With the continuous disparities between blacks and whites on CaP incidence, survival, and deaths, a key way to close the gap is individual health promotion and disease prevention behaviors to reduce the behavioral risk factors for CaP.

### Prostate cancer health beliefs

One of the potential sources of disparities in CaP is due to the variability in individual health promotion and disease prevention behaviors. [3] Thus, individual behavioral theories such as the Health Belief Model, [4,5] play a significant role in predicting, explaining and modifying health behaviors including CaP prevention, information-seeking and screening behaviors. The Health Belief Model postulate that an individual’s behavior is affected by perceptions of the threat posed by a health problem, the benefits of avoiding the threat, and factors influencing the decision to act. [4,5] Some of the key health belief concepts include: (1) Perceived susceptibility - opinion of chances of getting CaP; (2) Perceived severity - opinion about the seriousness of CaP and its sequel; (3) Perceived benefits - opinion about the benefits of reducing the risk of getting CaP; (4) Perceived barriers - opinion about the barriers impacting CaP prevention/early detection behaviors; (5) Cues to action - strategies to inform about and activate CaP prevention/early detection; and (6) Perceived behavioral control - confidence of participants’ ability to reduce the risk of getting CaP. Health belief factors such as perceived barriers [6,7] and cues to action from health care provider [8] have been found to significantly determine CaP screening in past studies. Also, intention to participate in CaP screening was found to be determined by perceived behavioral control, perceived susceptibility, and cues to action by health care provider.[9] Recently, our research team found modifiable CaP risk reduction behavior to be influenced by perceived severity, cues to action, knowledge, and behavioral control, while CaP detection behavior (information-seeking and screening) was determined by perceived susceptibility, attitude, perceived behavioral control, knowledge, and acculturation.[10]

### Prostate cancer cultural beliefs

A key piece currently missing in the CaP literature is the role of varying personal cultural beliefs and values on individual behavior among black men. It has been noted that fundamental elements related to ethnicity and culture shape health perceptions, attitudes and behaviors.[11] It is thus important to acknowledge cultural diversity and study the specific cultural beliefs of each ethnic group as related to health and health behaviors. [11] The importance of culture in the Black community has been recognized by researchers, [12-15] including the US Department of Health and Human Services. [14] Thereby, knowing the cultural worldview of black men will further improve our understanding of CaP prevention and control behaviors, [16] and ultimately enhance the design of successful interventions.

According to Leininger, [17] culture is defined as shared beliefs, values, customs, behaviors and artifacts used by individuals within a society to cope with other people and the world in general, and passed down from one generation to another through learning. The cultural worldview of individuals is rooted in the values, beliefs and behaviors of their ethnic population. [18,19] Cultural beliefs and values such as cancer fatalism, religion and spiritualism, temporal orientation and acculturation consequently affect their health beliefs, assumptions and behavior. *Cancer fatalism*, defined as an individual’s belief that death is bound to happen when diagnosed with cancer, is a major barrier to cancer detection and control. [20] Among blacks, fatalistic perspectives have been reported to affect cervical cancer, [21] breast cancer, [22,23] colorectal cancer[24] and fecal occult blood testing. [25] Although reports on the impact of *religion and spiritualism* on cancer prevention or screening is limited, it has been suggested that it may deter women from seeking treatment for breast cancer. [26]*Temporal orientation* describes the role of social psychology of time. It is an individual’s perception of time as being in the past, present or future[27,28] and has significant influence on individual thoughts and actions. [29] For example, temporal orientation has been found to predict mammography screening [23] and participation in genetic risk assessment. [30] In general, health promotion and disease prevention behaviors such as CaP prevention and screening require future time perspective. [29] It is interesting to note that Blacks’ time orientation has been reported to be in the present. [27,31,32] This is likely to have negative consequences on black men’s CaP prevention behavior. *Acculturation* is a cross-cultural psychology concept that “reflects the extent to which individuals (from a non-dominant culture) learn the values, behaviors, lifestyles, and language of the host (dominant) culture. [33] Since black men belong to a non-dominant group in the US, over the years they will adopt values, behaviors and lifestyles of the dominant Caucasian group. The level and process of acculturation differs for each individual and are likely to influence CaP health behaviors.

Published literature report on the CaP health beliefs of black men is limited and we are unaware of any published study reporting the cultural beliefs of black men relative to CaP. Thus, the goal of this paper was to explore the health beliefs and cultural beliefs for CaP among black men, and the key socio-demographic correlates of these beliefs. The health beliefs examined were perceived susceptibility, perceived severity, attitude, outcomes beliefs, and perceived behavioral control. This paper also examined the following cultural belief factors: CaP fatalism, temporal orientation, religiosity, and acculturation. We hypothesized that these beliefs will vary by socio-demographic characteristics such as age, education, marital status and ethnicity within the black race.

## Methods

The Florida Prostate Cancer Disparity project is a cross-sectional study of over 3,000 Black men in Florida to develop a *Personal Integrative Model of Prostate Cancer Disparity* (PIPCaD model). [10] A cross-sectional survey study design was employed to collect data between April 2008 and October 2009 from black men between the ages of 40 and 70 years. The comprehensive details of the methodology for this study have already been provided in previous publications. [10,34]

### Participants

The primary study sites for the study were Tallahassee, Miami, Tampa Bay, Jacksonville and Orlando cities in Florida. These cities were selected based on the large number of ethnic diversity of black men (i.e. U.S.-born and foreign-born). The study targeted black men, regardless of country of origin, between the age of 40 and 70 years. Although data were collected from all black men regardless of personal history of CaP, only the men who reported no personal history of CaP were included in the final data analyses.

An aggressive recruitment campaign was launched by our academic-community research team to recruit a demographically representative sample of black men through: barber shops, local black churches, mosques, community pharmacies, fraternities and social organizations (First Fridays, 100 Black men organizations), and radio/newspaper advertisements in the black media. The primary data collection sites were at ethnic barber shops and organized health events by community-based/ faith-based organizations.

### Measures

The study independent variables were ***health belief factors*** (perceived susceptibility, perceived severity, attitude outcomes beliefs, and perceived behavioral control) and ***cultural belief/value factors*** (cancer fatalism, temporal orientation, religiosity/ spiritualism, and acculturation). The health belief measures were previously developed by our research team and found to be reliable and valid. [9,10,34-36] For the cultural belief and value measures, the survey items were developed from: (i) findings of our research team from an ethnographical study of Black men’s cultural beliefs and values; [10] and (ii) the adaptation of the following measures: acculturation scale of Klonoff and Landrine, [37] Brown and Segal’s Hypertension Temporal Orientation scale, [31] Powe’s Fatalism Inventory, [24,25] and the Religious Coping scale by Carver et al. [38] The operational definition of the study variables are provided below.

#### Perceived susceptibility

Perceived susceptibility comprised three items on participants’ chances of getting CaP disease, with responses ranging from strongly agree (5) to strongly disagree (1). One of the items was: “There is a good possibility that I will get CaP.” Higher score for this variable indicated high perception of susceptibility to CaP disease. The a-priori scoring classification for this scale was low susceptibility for scores of 3-7, moderate susceptibility for scores of 8-11, and high susceptibility for scores of 12-15.

#### Perceived severity

The measure for this variable comprised three items about the seriousness and consequences of CaP. For example, participants responded to the statement: “If I had CaP, my whole life would change.” The scale score ranged from 1 (strongly disagree) to 5 (strongly agree), with higher score indicating high perceived severity. The a-priori scoring classification was low severity for scores of 3-7, moderate severity for scores of 8-11, and high severity for scores of 12-15.

#### Attitude

Attitude is the positive or negative evaluations about CaP. Five items measured participants’ attitude towards CaP screening, CaP risk reduction, and participating in CaP medical research on a very favorable/very unfavorable scale with higher score indicating positive attitude. An example of the attitude items was: “Getting tested for CaP with the Digital Rectal Examination (DRE) every year is:”. The a-priori scoring classification for this scale was low attitude for scores of 5-12, moderate attitude for scores of 13-19, and high attitude for scores of 20-25.

#### Outcome beliefs

This is defined as the beliefs about the outcomes (negative or positive) of a behavior such as CaP prevention and screening. Participants responded to four items on a strongly disagree (1) - strongly agree (5) response scale with higher score indicating positive outcome beliefs. An example of the outcome belief items is: “Preventing CaP through activities such as eating right, taking supplements and exercising will save my life.” The a-priori scoring classification for this scale was negative outcome beliefs for scores of 4-9, neutral outcome beliefs for scores of 10-15, and positive outcome beliefs for scores of 16-20.

#### Perceived behavioral control

Perceived behavioral control is the confidence of participants’ ability relative to enacting the behavior. Five items were used to assess how easy or difficult it was for respondents to participate in CaP screening, CaP risk reduction, and CaP medical research on a scale of 1 (very difficult) to 5 (very easy) with higher score indicating high perceived behavioral control. For example, participants responded to the statement: “Eating right and taking supplements to prevent CaP is:”. The a-priori scoring classification for this scale was low perceived behavioral control for scores of 5-12, moderate perceived behavioral control for scores of 13-19, and high perceived behavioral control for scores of 20-25.

#### Acculturation

The measure of acculturation was based on the acculturation scale of Klonoff & Landrine [37] and included items such as, “When it comes to the music I listen to and the movies I watch, they are mostly by African American artists” on a response scale ranging from strongly disagree (1) to strongly agree (5). High score on the acculturation scale indicated low level of acculturation, i.e. low adoption of the values, behaviors and lifestyles of others. The a-priori scoring classification for this scale was high acculturation for scores of 4-9, moderate acculturation for scores of 10-15, and low acculturation for scores of 16-20.

#### Temporal orientation

The three items used to assess temporal orientation were based on Brown and Segal’s [27] Hypertension Temporal Orientation scale. For example, participants responded to the statement: “I only live for now and will not worry about screening for CaP or preventing CaP.” A strongly disagree (1) to strongly agree (5) response scale was employed to capture participants’ responses. Low score on this measure indicated future time perspective. The a-priori scoring classification for this scale was high future time perspective for scores of 3-7, moderate future time perspective for scores of 8-11, and low future time perspective for scores of 12-15.

#### Prostate Cancer fatalism

Individual’s belief that death is bound to happen when diagnosed with cancer is a major barrier to cancer detection and control. [20] The measure of CaP fatalism was adapted from the Powe Fatalism Inventory. [24,25] Participants responded to three items on a strongly disagree (1) to strongly agree (5) response scale. One of the items was “I believe that if someone has CaP, it is already too late to do something about it”. A high score on this scale is an indication of strong belief that death is bound to happen when diagnosed with CaP. The a-priori scoring classification for this scale was low fatalism for scores of 3-7, moderate fatalism for scores of 8-11, and high fatalism for scores of 12-15.

#### Religiosity

Religiosity is organized system of beliefs, practices, rituals, and symbols. [39] Carver et al.’s scale[38] was adapted for the religious coping measures and included items such as: “I usually put my trust in God” and “My spirituality will help me to deal with any health problem.” The scale score ranged from 1 (strongly disagree) to 5 (strongly agree), with higher score indicating high religiosity. The a-priori scoring classification for this scale was low religiosity for scores of 3-7, moderate religiosity for scores of 8-11, and high religiosity for scores of 12-15.

### Data Collection

Data were collected by trained research assistants who provided the study surveys to black men after they provided consent to participate in the study. The survey was in the English language and was self-administered by participants. Upon completion of the surveys, participants were provided a $15 gift certificate towards a haircut at participating barber shops or a $10 Wal-Mart gift card as incentive for their participation.

### Analyses

The analyses of survey responses were conducted using the PC-SAS analytical software after the data coding and entry. The statistical analyses included frequency analysis of the variables to confirm responses were appropriately entered and to correct any errors, and the internal consistency of the study measures to establish the reliability of the measures. Subsequently, descriptive statistics were employed to summarize socio-demographic and study variables. Finally, multiple linear regression analyses were conducted to confirm the key socio-demographic correlates of CaP health and cultural beliefs.

## Results

A total of 2,864 responses were found to be complete and valid for this study. The study scales were found to be reliable based on Nunnaly’s[40] suggestion of 0.7 to be an acceptable reliability coefficient. The reliability (alpha) of the study measures were: 0.87 for attitude; 0.83 for perceived behavioral control; 0.87 for perceived susceptibility; 0.85 for perceived severity; 0.93 for outcome beliefs; 0.71 for acculturation; 0.85 for temporal orientation; 0.92 for cancer fatalism; and 0.91 for religiosity.

The characteristics of study participants are summarized in Additional file 1. Majority of the study participants were US-born black men, between 40 and 49 years, married, had full-time employment, at an education level of high school diploma, and earned less than $20,000.

### Health and cultural beliefs of participants

Additional file 2 provides a summary of the health beliefs and cultural beliefs of participants. The men’s overall attitude was favorable with a median of 20 on a scale ranging from 5 to 25. For each of the following items on the attitude scale, the median was 4 (i.e., favorable attitude): (1) Getting tested for CaP with the DRE every year; (2) Getting tested for CaP using Serum Prostate Specific Antigen (PSA) Test every year; (3) Doing activities such as, eating right and taking supplements, to prevent CaP; (4) Doing activities such as, exercising, to prevent CaP; and (5) Participating in CaP medical research.

The overall perceived behavioral control for the men was moderate, with a median of 19 on a scale range of 5 - 25. It was interesting to note that while the men’s responses indicated that it was easy for them to participate in CaP screening by PSA, eat right and take supplement, and exercise; their median responses were neutral (neither easy or difficult) for DRE every year and participating in CaP medical research. Relative to outcome beliefs, participants generally had positive outcome beliefs and agreed that: (1) Preventing CaP through activities such as eating right, taking supplements and exercising will save their lives; (2) Preventing CaP through activities such as eating right, taking supplements and exercising will help them to prevent other diseases; (3) Screening for CaP every year will allow them to detect the disease early and get appropriate treatment on time; and (4) Getting screened for CaP every year will give them peace of mind. On the other hand, the black men’s perception of CaP susceptibility (median of 9 on a scale range of 3-15) and perceived CaP severity (median of 11 on a scale range of 3-15) were found to be moderate.

The median score for acculturation was 16 (scale range of 4-20), 6 for temporal orientation (scale range of 3-15), 6 for cancer fatalism (scale range of 3-15), and 13 for religiosity (scale range of 3-15). Based on the a-priori scoring classifications for these scales, the men had low level of acculturation, did not hold fatalistic beliefs about cancer, had high religious coping skills and had high future time perspective.

### Demographic correlates of health and cultural beliefs

Additional file 3 summarizes the multiple linear regressions results for the demographic correlates of participants’ health beliefs and cultural beliefs. The demographic correlates of perceived susceptibility were ethnicity and age. The individual coefficients for the correlates indicated that US-born black men'sÂ perceived susceptibility score is significantly higher than Caribbean-born black men. Ethnicity, age and income were found to be associated with perceived severity. US-born and African-born black men reported higher perceived severity compared to Caribbean-born black men. In addition, participants who earned more than $100,000 annually reported higher perceived severity compared to those who earned less than $100,000 annually.

The correlates of attitude were ethnicity, age, education, income and insurance. The following were the findings from the individual coefficient estimates (not shown in Additional file 3): US-born black men and African-born US citizens had more favorable attitude compared to Caribbean-born black men; Black men between 60 and 69 years had more favorable attitude compared to the men between 40 and 49 years; Black men who did not complete high school and those with high school diploma had less favorable attitude compared to those with post-college degrees; Black men who earned $100,000 or more annually had more favorable attitude compared to the men who earned between $20,000 and $39,999 annually; and Black men who had health insurance had more favorable attitude compared to those with no health insurance.

Ethnicity, education, marital status, income and insurance were associated with outcome beliefs. US-born black men had more positive outcome beliefs compared to Caribbean-born black men; Black men with high school diploma or lower had less positive outcome beliefs compared to those with post-college degrees; single men had less positive outcome beliefs compared to widowed men; Black men who earned $100,000 or more annually had more positive outcome beliefs compared to the men who earned between $20,000 and $39,999 annually; and black men who had health insurance had more positive outcome beliefs compared to those without health insurance. The demographic correlates of perceived behavioral control were age, education, income and insurance. The following were the findings from the individual coefficient estimates: Black men between 60 and 69 years reported higher perceived behavioral control compared to the men between 40 and 49 years; Black men with some college training, high school diploma and less than high school education reported lower perceived behavioral control compared to those with post-college degrees; and black men with health insurance reported higher perceived behavioral control compared to those without health insurance.

For the cultural beliefs/values factors, the demographic correlates were: income and insurance for acculturation; ethnicity, education, marital status, employment status, income and insurance for both temporal orientation and cancer fatalism; and ethnicity, education, income and insurance for religiosity. Participants who reported a household income less than $60,000 were less acculturated compared to those who reported annual income of $100,000 or more. Black men who had health insurance were less acculturated compared to those with no health insurance. Relative to temporal orientation: US-born black men were more future oriented compared to Caribbean-born black men; Black men with high school diploma or lower education were less future-oriented compared to those with post-college degrees; men who reported being retired were more future-oriented compared to unemployed men; men who earn $100,000 or more were more future-oriented compared to the men who earn less than $80,000 annually; and men with health insurance were more future-oriented compared to the men without health insurance.

For cancer fatalism: US-born black men and Caribbean-born US citizens reported less cancer fatalism compared to Caribbean-born black men; Black men with high school diploma or lower education were reported higher cancer fatalism compared to those with post-college degrees; men with part-time employment reported higher cancer fatalism compared to unemployed men; men who earned less than $80,000 annually reported higher cancer fatalism compared to men who earned $100,000 or more annually; and men without health insurance reported higher cancer fatalism compared to those with health insurance.

Finally, ethnicity, education, income and insurance were found to be associated with religiosity. US-born black men reported higher level of religiosity compared to Caribbean-born black men, while men without health insurance reported lower level of religiosity compared to the men with health insurance.

## Discussion

To date, the disparate burden of CaP in black men is still poorly understood and has not been effectively addressed. Although black men may have several biological factors contributing to the higher incidence of CaP, the potential sources of CaP disparity occur at individual (personal or provider), and institutional or health systems levels. [3] Since behavioral factors is a significant determinant of population health, [41] addressing CaP-related health beliefs and cultural beliefs are key weapons to fight CaP.

In a recent study, black men’s CaP prevention behavior was found to be influenced by perceived severity and perceived behavioral control, and CaP detection (including information-seeking and screening behaviors) determined by perceived susceptibility, attitude, perceived behavioral control, and acculturation. [10] The confirmed associations among CaP health behaviors, health beliefs, and cultural beliefs underscore the importance of health beliefs and cultural beliefs in the promotion of CaP risk reduction behavior as well as early detection among black men.

Our current study found low perceived behavioral control among younger men, men who were not college-educated, and men without health insurance. Black men who earned less than $100,000 annually reported low perceived severity for CaP. The knowledge of these personal factors provides key information to develop tailored and targeted CaP educational interventions for black men. Based on our findings, men with low socioeconomic status and men less than 50 years reported low confidence in their ability to reduce their risk of getting CaP and early detection of CaP. Educational interventions should therefore focus on teaching them how to eat healthy to prevent CaP and how to access accurate information to make informed decision about CaP screening.

An interesting finding is the lower perception of CaP susceptibility and severity among Caribbean-born black men compared to US black men. In another study, Odedina et al. [34] found within-group differences between native-born and foreign-born US black men on CaP risk reduction and early detection practices. Risk reduction behaviors (such as reduced meat consumption and use of chemoprevention) and self-initiated CaP discussion with a doctor was lower in native-born black men, although they were better insured and had higher CaP knowledge compared to African-born and Caribbean-born US black men. [34] Future studies should focus on the differential effect of US nativity and immigration status on CaP health disparities among black men. The study of migration and health will enhance our understanding of CaP etiology among Blacks and also foster better understanding of health risks among Black immigrants.

Another important contribution of this study is the exploration of socio-cultural experiences such as cultural beliefs and values. Currently, there is limited published study that has explored the role of black men’s cultural worldview on CaP risk reduction and detection behaviors. The participants of this study reported low acculturation, low cancer fatalism, high religious coping skills and high future time perspective. Interestingly, we found differential effect of foreign born on cancer fatalism, religiosity and temporal orientation. Given that previous studies have also noted health variations between US born and foreign born Blacks, with a health advantage proposed for US Black immigrants, [42-46] more research is needed to clarify if and how cultural beliefs and values impact CaP prevention and early detection. As suggested by the IOM report, [3] our premise is that the health behavior of black men contributes to CaP health disparity. By confirming the cultural beliefs and values that affect this behavior, targeted programs can be developed to promote CaP prevention and detection among black men.

The primary limitations of this study are inherent in the representation of participants and the nature of the study design. Since participants were convenient sample, study results cannot be readily generalized to all black men. Another limitation is that the assessment of study variables is by self-administered survey which may be biased by social desirability (lying to look good), acquiescence (tendency to agree), and extremity (tendency to use extreme ratings).

## Competing interests

The authors declare that they have no competing interests.

## Contribution to literature

Our study provides rich data with regard to the health and cultural beliefs that might serve to inform the development of cancer control initiatives, including CaP prevention, CaP screening and CaP education programs in these high risk target populations.

## Supplementary Material

Additional file 1 Participants’ demographics Click here for file

Additional file 2 Summary of health beliefs and cultural beliefs of participants Click here for file

Additional file 3 Multiple regression analyses results for demographic correlates Click here for file

## References

[B1] American Cancer Society Cancer Facts & Figures for African Americans 2009-2010 2009 American Cancer Society. Atlanta, GA

[B2] American Cancer Society Cancer Facts & Figures 2010 2010 American Cancer Society. Atlanta, GA

[B3] Institute of Medicine Smedley BD, Stith AY, Nelson AR Unequal treatment: Confronting racial and ethnic disparities in healthcare 2003 Washington DC, National Academy Press 1 27 25032386

[B4] Rosenstock IM Historical origins of the health belief model Health Educ Monogr 1974 2 344 10.1177/109019817800600406 299611

[B5] Becker MH Maiman LA Kirscht JP The health belief model and prediction of dietary compliance: a field experiment J Health Soc Behav 1997 18 348 366 617639

[B6] Shane L Wilson T Prostate cancer, income and education among Afro-Americans in Florida McNair Journal 1999 39 42

[B7] Shelton P Weinrich S Reynolds WA Jr Barriers to prostate cancer screening in African American men J Natl Black Nurses Assoc 1999 10 2 14 28 10732593

[B8] Nivens AS Herman J Weinrich SP Cues to participation in prostate cancer screening: a theory for practice Oncol Nurs Forum 2001 28 9 1449 1456 11683314

[B9] Odedina FT Campbell E Scrivens J Emanuel A LaRose-Pierre M Brown J Nash R Personal Factors Affecting African American men’s prostate cancer screening behavior J Natl Med Assoc 2008 100 6 724 733 PMID: 18595577 18595577 10.1016/s0027-9684(15)31350-x

[B10] Odedina FT Scrivens J LaRose-Pierre M Emanuel A Adams AA Gagne GA Pressey SA Odedina AO Modifiable Prostate Cancer Risk Reduction and Early Detection Behaviors in Black Men Am J Health Behav 2011 in press Forthcoming 10.5993/ajhb.35.4.9 22040593

[B11] Leininger M Nursing theories and culture: Fit or misfit? J Transcult Nurs 1995 7 1 41 42 10.1177/104365969500700107 8716105

[B12] Harrison I Harrison D Crawford C The black family experience and health behavior 1971 Health and the Family: A medical-sociological analysis. New York: Macmillan 171 199

[B13] Hogle J Ethnicity and utilization of health services: An urban response to a Community Health Center 1982 Ann Arbor, Michigan: University Microfilms International

[B14] US Department of Health and Human Services Report of the Secretary’s Task Force: Black and Minority Health, Executive Summary 1985 1 Government Printing Office 187 194

[B15] Bailey EJ Sociocultural factors and health care-seeking behavior among Black Americans J Natl Med Assoc 1987 79 4 389 392 3586036 PMC2625488

[B16] Hughes C Fasaye GA LaSalle VH Finch C Sociocultural influences on participation in genetic risk assessment and testing among African American women Patient Educ Couns 2003 51 107 114 10.1016/S0738-3991(02)00179-9 14572939

[B17] Leininger M Nursing and anthropology: Two worlds to blend 1970 New York: John Wiley & Sons

[B18] Myers LJ Understanding and Afrocentric worldview: Introduction to an optimal psychology 1998 Dubuque, IA: Kendall-Hunt

[B19] Jackson AP Sears SJ Implications of an Afrocentric worldview in reducing stress for African American women J Couns Dev 1992 71 184 190

[B20] Powe BD Finnie R Cancer fatalism: The state of the science Cancer Nurs 2003 26 454 467 15022977 10.1097/00002820-200312000-00005

[B21] Chavez LR Mishra SI Hubbell FA Valdez RB The influence of fatalism on self reported use of papanicolaou smears Am J Prev Med 1997 13 418 424 9415785

[B22] Mayo RM Ureda JR Parker VG Importance of fatalism in understanding mammography screening in rural elderly women J Women Aging 2001 13 57 72 11217186 10.1300/J074v13n01_05

[B23] Russell KM Perkins SM Zollinger TW Champion VL Sociocultural context of mammography screening use Oncol Nurs Forum 2006 33 1 105 112 10.1188/06.ONF.105-112 16470238

[B24] Powe BD Fatalism among elderly African Americans: Effects on colorectal cancer screening Cancer Nurs 1995 18 285 392 7585493

[B25] Powe BD Cancer fatalism among elderly Caucasians and African Americans Oncol Nurs Forum 1995 22 9 1355 1359 8539176

[B26] Lannin DR Mathews HF Mitchell J Influence of socioeconomic and cultural factors on racial differences in late-stage presentation of breast cancer JAMA 1998 279 1801 1807 10.1001/jama.279.22.1801 9628711

[B27] Brown CM Segal R The development and evaluation of the hypertension temporal orientation (HTO) scale Ethn Dis 1997 7 41 54 9253555

[B28] Holman EA Silver RC Getting stuck in the past: Temporal orientation and coping with trauma J Pers Soc Psychol 1998 74 1146 1163 9599436 10.1037//0022-3514.74.5.1146

[B29] Graham RJ The role of perception of time in consumer research J Consum Res 1981 7 335 342 10.1086/208823

[B30] Hughes C Fasaye GA LaSalle VH Finch C Sociocultural influences on participation in genetic risk assessment and testing among African American women Patient Educ Couns 2003 51 107 114 10.1016/S0738-3991(02)00179-9 14572939

[B31] Brown CM Segal R Ethnic differences in temporal orientation and its implications for hypertension management J Health Soc Behav 1996 37 350 361 10.2307/2137262 8997890

[B32] Jones JM McGrath JE Cultural differences in temporal perspectives: Instrumental and expressive behaviors in time The Social Psychology of Time 1988 Newbury Park, Calif: Sage Publications 21 38

[B33] Zane N Mak W Chun KM, Organista PB, Marin G Major approaches to the measurement of acculturation among ethnic minority populations: A content analysis and an alternative empirical strategy Acculturation: Advances in Theory, Measurement, and Applied Research 2003 Washington, DC: American Psychological Association 39

[B34] Odedina FT Gagne GA LaRose-Pierre M Emanuel A Scrivens J Adams AA Pressey SA Odedina AO Within-group differences between native-born and foreign-born Black men on prostate cancer risk reduction and early detection practices J Immigr Minor Health 2011 in press Forthcoming 10.1007/s10903-011-9471-8 21547350

[B35] Odedina FT Scrivens J Xiao H African American males' views on prostate cancer screening Minority Health 2000 1 6 28 34

[B36] Odedina FT Scrivens J Emanuel A A focus group study of factors influencing African-American men's prostate cancer screening behavior J Natl Med Assoc 2004 96 6 780 788 15233488 PMC2568374

[B37] Klonoff EA Landrine H Revising and improving the African American Acculturation scale J Black Psychol 2000 26 2 235 261 10.1177/0095798400026002007

[B38] Carver CS Scheier MF Weintraub JK Assessing coping strategies: a theoretically based approach J Pers Soc Psychol 1989 56 267 283 2926629 10.1037//0022-3514.56.2.267

[B39] Thoresen CE R.S. Roth & S.R. Kurpius Spirituality, Health, and science: the coming revival? The Emerging Role of Counseling Psychology in Health Care 1998 New York: W.W. Norton

[B40] Nunnaly J Psychometric Theory 1978 New York: McGraw-Hill

[B41] McGinnis JM Williams-Russon P Knickman JR The case for more active policy attentions to health promotion Health Aff 2002 21 2 78 92 10.1377/hlthaff.21.2.78 11900188

[B42] Read JG Emerson MO Tarlov A Implications of black immigrant health for U.S. racial disparities in health J Immigr Health 2005 7 3 205 212 10.1007/s10903-005-3677-6 15900421

[B43] Pallotto EK Collins JW Jr David RJ Enigma of maternal race and infant birth weight: A population-based study of US-born Black and Caribbean-born Black women Am J Epidemiol 2000 151 11 1080 1085 10873132 10.1093/oxfordjournals.aje.a010151

[B44] Schmidley AD Profile of the foreign-born population in the United States: 2000 U.S. Census Bureau, Current Population Reports, Series 2001 Washington, DC: U.S. Government Printing Office 23 206

[B45] Singh GK Siahpush M Ethnic-immigrant differentials in health behaviors, morbidity, and cause-specific mortality in the United States: An analysis of two national data bases Hum Biol 2002 74 83 109 10.1353/hub.2002.0011 11931581

[B46] David RJ Collins JW Differing birth weight among infants of U.S.-born blacks, African-born blacks and U.S.-born whites N Engl J Med 1997 337 17 1209 1214 10.1056/NEJM199710233371706 9337381

