# Supplementary material for: Prostate cancer health and cultural beliefs of black men: The Florida Prostate Cancer Disparity Project
Source: Infect Agent Cancer. 2011 Sep 23;6(Suppl 2):S10. doi: 10.1186/1750-9378-6-S2-S10 (PMC3194180; doi:10.1186/1750-9378-6-S2-S10)
Supplement: Additional file 1 — Participants’ demographics [file 1750-9378-6-S2-S10-S1.pdf]

Table 1
Participants' Demographics

**Variable**	Frequency	**Percent (%)**
Ethnicity		
African American of American origin	2066	79.86
African American of African origin	145	5.60
African American of Caribbean origin	153	5.91
African	108	4.17
Caribbean	115	4.45
Frequency Missing	277	
Age		
40 to 49	1365	50.15
50 to 59	971	35.67
60 to 69	386	14.18
Frequency Missing	142	
Education		
Less than high school	342	12.84
High school degree	1046	39.28
Some college training	540	20.28
College degree	531	19.94
Post-college degree	204	7.66
Frequency Missing	201	12.84
Marital Status		
Single	1057	38.53

Married	1279	46.63
Divorced	333	12.14
Widowed	74	2.70
Frequency Missing	121	
Employment		
Full time	1333	48.83
Part time	312	11.43
Disability	252	9.23
Retired	251	9.19
Unemployed	582	21.32
Frequency Missing	134	
Household Income ($)		
0-19,999	1052	39.30
20,000-39,999	659	24.62
40,000-59,999	360	13.45
60,000-79,999	24	9.15
80,000 – 99,999	5	5.90
100,000 and above	158	7.58
Frequency Missing	203	
Insurance		
Yes	1710	63.81
No	970	36.19
Frequency Missing	184	
