# Supplementary material for: Prostate cancer health and cultural beliefs of black men: The Florida Prostate Cancer Disparity Project
Source: Infect Agent Cancer. 2011 Sep 23;6(Suppl 2):S10. doi: 10.1186/1750-9378-6-S2-S10 (PMC3194180; doi:10.1186/1750-9378-6-S2-S10)
Supplement: Additional file 2 — Summary of health beliefs and cultural beliefs of participants [file 1750-9378-6-S2-S10-S2.pdf]

Table 2

Summary of Health Beliefs and Cultural Beliefs of Participants

**Study Variables**	**Scale Range**	**Mean (SD)**	**Median**
Perceived susceptibility	3- 15	9.23(2.84)	9
Perceived severity	3- 15	10.35 (2.95)	11
Attitude	5- 25	19.64 (4.08)	20
Perceived Behavioral Control	5- 25	18.46 (3.98)	19
Acculturation	4- 20	15.42 (3.16)	16
Temporal Orientation	3- 15	7.09 (3.29)	6
Cancer Fatalism	3-15	6.35 (3.29)	6
Religiosity	3- 15	12.47 (2.96)	13
