# Supplementary material for: Prostate cancer health and cultural beliefs of black men: The Florida Prostate Cancer Disparity Project
Source: Infect Agent Cancer. 2011 Sep 23;6(Suppl 2):S10. doi: 10.1186/1750-9378-6-S2-S10 (PMC3194180; doi:10.1186/1750-9378-6-S2-S10)
Supplement: Additional file 3 — Multiple regression analyses results for demographic correlates [file 1750-9378-6-S2-S10-S3.pdf]

Table 3

Multiple Regression Analyses Results for Demographic Correlates

**Variable / Item**	**F-Values and Probability Values (Pr > F) for**						
	**Ethnicity**	**Age**	**Education**	**Marital Status**	**Employment status**	**Income**	**Insurance**
Perceived susceptibility	**4.64 (0.0010)**	**3.99 (0.0186)**	0.91 (0.4570)	2.35 (0.0706)	0.38 (0.8213)	0.97 (0.4357)	2.21 (0.1369)
Perceived severity	**3.02 (0.0171)**	**3.21 (0.0404)**	0.60 (0.6621)	2.21 (0.0854)	1.46 (0.2109)	**2.67 (0.0205)**	1.36 (0.2438)
Attitude	**3.23 (0.0119)**	**16.03 (<.0001)**	**10.64 (<.0001)**	1.84 (0.1382)	1.73 (0.1413)	**6.71 (<.0001)**	**5.60 (0.0180)**
Outcome Beliefs	**7.46 (<.0001)**	2.31 (0.0992)	**9.88 (<.0001)**	**2.91 (0.0332)**	2.23 (0.0630)	**3.62 (0.0029)**	**8.94 (0.0028)**
Perceived behavioral control	0.81 (0.5206)	**12.46 (<.0001)**	**7.83 (<.0001)**	1.88 (0.1317)	1.77 (0.1324)	**2.31 (0.0421)**	**10.34 (0.0013)**
Acculturation	0.77 (0.5455)	0.94 (0.3908)	1.51 (0.1965)	0.74 (0.5255)	0.37 (0.8316)	**4.30 (0.0007)**	**8.86 (0.0029)**
Temporal orientation	**6.02 (<.0001)**	1.23 (0.2935)	**13.04 (<.0001)**	**7.19 (<.0001)**	**2.60 (0.0342)**	**5.93 (<.0001)**	**14.92 (0.0001)**
Cancer fatalism	**8.69 (<.0001)**	0.34 (0.7129)	**9.26 (<.0001)**	**5.13 (0.0016)**	**3.18 (0.0130)**	**4.39 (0.0006)**	**9.68 (0.0019)**
Religiosity	**5.66 (0.0002)**	0.70 (0.4988)	**3.12 (0.0143)**	1.88 (0.1303)	1.24 (0.2920)	**2.47 (0.0308)**	**29.67 (<.0001)**

† Results in bold-font are statistically significant at p<0.05 level.
